# Visible and Near-Infrared Spectroscopy Analysis of a Polycyclic Aromatic Hydrocarbon in Soils

**DOI:** 10.1155/2013/160360

**Published:** 2013-12-25

**Authors:** Reuben N. Okparanma, Abdul M. Mouazen

**Affiliations:** Department of Environmental Science and Technology, Cranfield University, Cranfield, Bedfordshire MK43 0AL, UK

## Abstract

Visible and near-infrared (VisNIR) spectroscopy is becoming recognised by soil scientists as a rapid and cost-effective measurement method for hydrocarbons in petroleum-contaminated soils. This study investigated the potential application of VisNIR spectroscopy (350–2500 nm) for the prediction of phenanthrene, a polycyclic aromatic hydrocarbon (PAH), in soils. A total of 150 diesel-contaminated soil samples were used in the investigation. Partial least-squares (PLS) regression analysis with full cross-validation was used to develop models to predict the PAH compound. Results showed that the PAH compound was predicted well with residual prediction deviation of 2.0–2.32, root-mean-square error of prediction of 0.21–0.25 mg kg^−1^, and coefficient of determination (*r* ^2^) of 0.75–0.83. The mechanism of prediction was attributed to covariation of the PAH with clay and soil organic carbon. Overall, the results demonstrated that the methodology may be used for predicting phenanthrene in soils utilizing the interrelationship between clay and soil organic carbon.

## 1. Introduction

**Figure 1:**
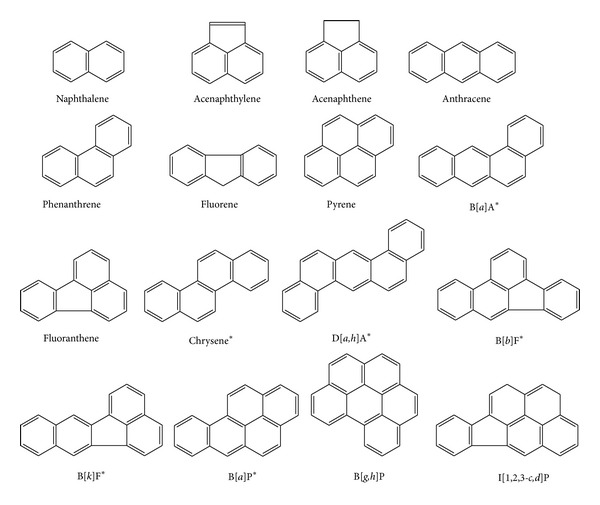
Chemical structures of the 16 United States Environmental Protection Agency (USEPA) priority PAH compounds. *Non-threshold indicator compounds, also known to possess some genotoxic carcinogenic potential [8]. B[*a*]A: benzo[*a*]anthracene; D[*a*,*h*]A: dibenzo[*a*,*h*]anthracene; B[*b*]F: benzo[*b*]fluoranthene; B[*k*]F: benzo[*k*]fluoranthene; B[*a*]P: benzo[*a*]pyrene; B[*g*,*h*]P: benzo[*g*,*h*,*i*]perylene; I[1,2,3-*c*,*d*]P: indeno[1,2,3-*c*,*d*]pyrene.

Polycyclic aromatic hydrocarbons (PAHs) are the class of hydrocarbons containing two or more fused aromatic hydrocarbons. PAHs are found in large quantities in mineral oil such as diesel fuel #2 (Chemical Abstract Service [CAS] no. 68476-34-6)—simply referred to as diesel. PAHs are harmful to the environment because some are potentially carcinogenic or mutagenic [1]. The United States Environmental Protection Agency (USEPA) has classified sixteen PAHs (including phenanthrene) as priority pollutants for investigating PAH pollution in the environment (Figure 1).

These priority PAHs have molecular mass ranging from 128 gmol^−1^ for naphthalene to 278 gmol^−1^ for dibenzo[*a*,*h*]anthracene. The solubility, sorption, and vapour pressure characteristics of PAHs are important factors that control their distribution in the environment [1, 2]. PAH compounds are known to exhibit very low water solubility. Only few are slightly soluble. Since PAHs exhibit strong hydrophobicity, they primarily sorb to the organic matter in the soil [2].

The concentration and compositional distribution of PAHs in an environmental sample such as soil are widely used to identify their origin or source by PAH diagnostic ratio analysis [3–5]. The nature and concentration of PAHs are vital in tackling their proliferation in the environment by informing risk assessment and remediation. Conventional methods of quantifying PAHs in contaminated soils such as gas chromatography-mass spectrometry (GC-MS) are highly sensitive and specific [6]. But, they are relatively expensive, involve time-consuming sample preparation protocols, and rely on the use of noxious extraction solvents that tend to pose a health risk to operators [7]. This has prompted increasing demand for alternative methods capable of overcoming most of those challenges, without necessarily having to trade off instrument performance, to complement the conventional methods.

One of these emerging alternatives is the VisNIR spectroscopy analysis. VisNIR prediction is based on overtones and combinations of fundamental vibrations occurring in the mid-infrared region. The difficulty in interpreting VisNIR spectra because of broad and overlapping bands [9], to a large extent, has been overcome by the use of advanced chemometrics and data-processing techniques [10]. When analysing spectroscopic data, multivariate calibration generally solves the problem of interference from compounds closely related to the target compound, thereby eliminating the need for selectivity [11]. The origin of VisNIR spectra of hydrocarbon derivatives is attributed to the combinations or overtones of C–H stretching modes of saturated CH_2_ and terminal CH_3_, or aromatic C–H (ArCH) functional groups [12]. The intensity and wavelength positions of the vibrating molecule typify the origin of the spectral feature and can be used to identify the properties of the substance by multivariate analytical techniques [10]. The total petroleum hydrocarbon (TPH) content in hydrocarbon-contaminated soils has been predicted by VisNIR spectroscopy using various multivariate techniques [13–19]. A recent review shows that the quality of these TPH models has improved recently, suggesting a greater likelihood of using VisNIR spectroscopy as a screening tool for hydrocarbon-contaminated soils [7]. It must be pointed out that because of the high variability in a sample or site matrix, more research is still necessary to achieve the required level of accuracy prescribed by the industry and/or regulatory agencies for an online manufacturing system. This is because the models reported so far appear to be local models developed for specific sampling sites or individual soil types. Nevertheless, these models may provide vital information for the development of regional or global model for general application. Moreover, only benzo[*a*]pyrene out of the sixteen priority PAHs has been predicted by VisNIR spectroscopy demonstrating good accuracy (average prediction accuracy = 78.9%) but moderate to high false-positive rates. [20]. The high false-positive rates reported for benzo[*a*]pyrene by Bray et al. [20] and the absence of literature for the remaining PAHs accentuate the need for further research on the application of the approach for the determination of other PAHs in soils. As stated earlier, proper knowledge of the nature and concentration of individual PAHs in soils is crucial to risk-based assessment and remediation of petroleum release sites.

The objective of the current study was to evaluate the prediction accuracy of phenanthrene in diesel-contaminated soils by VisNIR spectroscopy and PLS regression analysis. This was evaluated for soil samples of different textures at various moisture contents and oil concentrations.

## 2. Materials and Methods 

### 2.1. Sample Collection and Treatment

The soil samples (arenosols, cambisols, and luvisols) used in this study were collected from four different fields at the experimental farm of Cranfield University at Silsoe (52°00′ N, 0°26′ W) and one field at Duck End Farm at Wilstead (52°05′ N, 0°27′ W), all in Central Bedfordshire, UK. The prediction ability of the VisNIR measurement approach was tested with two batches of samples that were given different forms of preparations. The first batch consisting of five groups of samples was given various treatments including drying, sieving, and wetting before contamination with diesel (graded set). The second batch, which is the sixth group, was contaminated with diesel without drying and sieving. Each group consisted of 25 samples all taken from one field except for the sixth group that was constituted with 5 samples from each field. This sixth group was used as field-moist intact samples, representing, mixed moisture content set. In all, a total of 150 soil samples were prepared. Drying was done by oven-drying method at 105 ± 5°C for 24 h while sieving was done with a 2 mm mesh size. Wetting was done by adjusting the moisture content of the dried samples to 0 (dry), 5, 10, 15, and 20% (w/w) with an adjustable-volume pipette (Eppendorf UK Ltd., Stevenage, UK). In wetting the samples, 5 samples in each group were adjusted to the same moisture content. Samples were contaminated with 852, 1136, 1420, 1705, and 1989 mm^3^ of diesel with an adjustable-volume pipette. The expected concentrations of the added diesel were 30,000, 60,000, 90,000, 120,000, and 150,000 mg kg^−1^ of soil, respectively. Similarly, 5 samples in each group were contaminated with the same oil concentration. Water (graded set only) and diesel were then added to the samples. The weight of each sample was approximately 25 g.

### 2.2. Reference Laboratory Analysis of Soil Physicochemical Properties

Soil particle size distribution was determined by laser diffraction with a Mastersizer2000 (Malvern Instruments, Worcestershire, UK) coupled to a HydroMu dispersing unit (Malvern Instruments, Worcestershire, UK). We used the United States Department of Agriculture (USDA) soil textural classification scheme to determine the soil textural classes on the basis of percent clay, silt, and sand. Soil moisture content (w/w), on dry basis, was determined by the oven-drying method at 105 ± 5°C for 24 h. Soil organic carbon was determined by the standard operating procedures of the Cranfield University based on British Standard 7755 Section  3.8 : 1995 [22] with a Vario EL III Analyzer (Elementar Analysensysteme, Hanau, Germany) (see Table 1).

We extracted PAH compound from the diesel-spiked soil samples by the sequential ultrasonic solvent extraction method [23] with a mixture of dichloromethane (DCM) and hexane (1 : 1). Cleanup of the PAH extract was carried out with an ~0.6 g Florisil (Fisher Scientific Ltd., Loughborough, UK), on glass wool, microscale column prewashed with DCM. PAH analysis was carried out with a 6890N Network Gas Chromatographic System (Agilent Technologies Inc., USA) coupled to a 5973 Network Mass Selective Detector (MSD) (Agilent Technologies Inc., USA) operated at 70 eV in positive ion mode. PAH compound was quantified by the internal standard method. The instrument was calibrated beforehand with a 5-level calibration solution mix. The calibration solution mix was made up with a EPA 525 PAH Mix-A standard solution (Sigma-Aldrich Co. Ltd., Dorset, UK) and deuterated PAH internal standard solutions: naphthalene-d^8^, anthracene-d^10^, chrysene-d^12^, and perylene-d^12^ (Sigma-Aldrich Co. Ltd., Dorset, UK). Quantification of PAH was performed by integrating the peak at specific mass-to-charge ratio (*m*/*z*) by means of MSD ChemStation. Major hydrocarbon compounds were identified on the basis of their retention time and by comparing them to those of analytical standards. Matrix spikes, duplicates, solvent, and method blanks were also analysed as quality control samples.

### 2.3. Optical Scanning of Soil Samples

Diffuse reflectance spectra were taken from the soil samples with a mobile fiber-optic LabSpec2500 VisNIR spectrophotometer (350–2500 nm) (Analytical Spectral Devices Inc., USA) coupled to a high-intensity probe (Analytical Spectral Devices Inc., USA). The spectrophotometer has one Si array (350–1000 nm) and two Peltier-cooled InGaAs detectors (1000–1800 nm and 1800–2500 nm). Spectral sampling interval of the instrument was 1 nm across the entire spectral range. However, the spectral resolution was 3 nm at 700 nm and 10 nm at 1400 and 2100 nm. The high-intensity probe has a built-in light source made of a quartz-halogen bulb of 2727°C. The light source and detection fibres are assembled in the high-intensity probe enclosing a 35-degree angle. Before the soil samples were scanned, and at intervals of 30 min, white-referencing with a Spectralon disc of almost 100% reflectance was carried out to optimize the instrument. Scans were taken from the soil sample, tightly packed and leveled in a cuvette, at three equidistant positions, 120° apart. Each sample was scanned nine times, three times per spot, and averaged for spectral preprocessing and multivariate analysis.

### 2.4. Spectral Preprocessing

Spectral preprocessing aims to reduce spurious peaks that do not contain the physical or chemical information and to correct physical scatter effects [11]. Perceived noises at the extremes of the spectrum (i.e., at 350–449 nm and 2451–2500 nm) were removed because of low instrument sensitivity at these wavelengths. Spectral truncation was followed by smoothing by averaging the adjacent 5 nm wavelengths to reduce the impact of noise. Thus, the VisNIR wavelength for modeling was in the range 452–2450 nm consisting of 401 wavelengths. To remove the additive baseline shift, the spectra were transformed by the Savitzky-Golay first derivative of polynomial order of two and two smoothing points. This was implemented for all sample subsets using the Unscrambler 9.8 (CAMO Software AS, Oslo, Norway).

### 2.5. Partial Least-Squares (PLS) Regression Analysis

Before calibration, spectral reflectance (R) was transformed to the logarithm of the relative intensity (1/R) or absorption [11]. The PLS regression analysis is a bilinear modeling method where information in the original *x*-data is projected onto a small number of underlying (“latent”) variables called PLS components. The *y*-data are actively used in estimating the “latent” variables to ensure that the first components are those that are most relevant for predicting the *y*-variables. Interpretation of the relationship between *x*- and *y*-data is then simplified as this relationship is concentrated on the smallest possible number of components (latent variables, LV). More detailed information about the PLS can be found in Martens and Naes [24]. We used PLS regression analysis with full cross-validation to relate the variation in a single-component variable (e.g., PAH) to the variation in a multicomponent variable (e.g., wavelength) with Unscrambler 9.8 (CAMO Software AS, Oslo, Norway). In this study, two categories of PLS models were developed. In the first category, models were developed for each oil treatment level as well as the field moist samples. To do this, the VisNIR spectra of the graded samples were separated into subgroups of 25 spectra according to oil treatment levels regardless of the moisture content. Then, the 25 spectra and chemical variables were used together to develop a PLS regression model for each treatment level. This was to determine and compare the ability of the technique to predict PAH across the range of moisture contents and diesel concentrations used and under field-moist conditions. Field-moist PLS models were developed with the 25 field-moist spectra and chemical variables together. Up to twelve LVs were considered, and the optimal number of LVs for future predictions was determined on the basis of the number of factors at the first local minimum [11]. In the second category, a general model was developed to predict the PAH compound with the entire 150 samples. In this category, the entire dataset was randomly separated into calibration (76%) and prediction (24%) sets. The ratio of calibration/prediction samples was chosen to ensure that each sample subset (group) was equally represented in the prediction set by randomly choosing 6 samples from each sample subset. PLS regression analysis with full cross-validation was carried out with the calibration set. The prediction set was then used to test the prediction accuracy of the calibration model. During model calibration in the second model category, potential outliers were identified on the basis of their influence on the *X*-*Y* relationship. Spectra that differed from the reference by three times the standard deviation of the predicted residuals were removed from the calibration dataset [25]. Model quality was statistically analyzed by the root-mean-square error (RMSE) of cross-validation and prediction, residual prediction deviation (RPD) (i.e., the ratio of standard deviation of laboratory-measured sample concentration to the RMSE), and corresponding coefficient of determination (*r* ^2^) [11]. RPD was originally defined by Williams and Sobering [26]. Model prediction ability was categorised based on the following criteria: excellent (i.e., symbol A) if RPD > 2.0, almost good (symbol B) if 1.4 ≤ RPD < 2.0, and unreliable (symbol C) if RPD < 1.4 [27]. Category of prediction is the ability of PLS full-cross-validation analysis for parameter validation and prediction [27].

## 3. Results and Discussion

### 3.1. Calibration Models of Phenanthrene

Table 2 summarizes the statistics of the PLS models for phenanthrene in calibration dataset. As can be seen in Table 2, 90% of the PLS models (excluding the field-moist models) were classified as almost good to excellent prediction (cross-validation) ability for model parameters, with 70% of the models in the excellent category, signifying the possibility for quantitative applications [28]. The model developed for 90,000 mg kg^−1^ oil treatment level using first derivative transformed spectra was the best (RPD = 3.88) as compared to others (Table 2). Only 10% of the models were unreliable. There is a considerable difference in the value of *r* ^2^ between 120,000 mg kg^−1^ and others. This poor correlation is because sample concentrations are not uniformly distributed over the working concentration range of 0.02 to 2.80 mg kg^−1^ (table is not shown). For the field-moist intact soil samples (representing mixed moisture content samples), model prediction ability was also classified as excellent. This suggests that VisNIR spectroscopic method may be used for predicting PAH in soils without lengthy sample preparations. These results largely demonstrate that the VisNIR spectroscopic method may be a useful tool for evaluating PAH concentrations in soils. Nevertheless, the success of the methodology in broader environmental applications will depend on a number of factors including (among others) the accuracy (i.e., extraction efficiency) of the reference analytical method and weathering process of petroleum products in soils over time. These two factors explain why, for instance, doubling the amount of added diesel oil does not double the PAH concentrations as one would expect (Table 2). In connection with providing information for risk assessment and remediation of contaminated soils, further research is required to ascertain the prediction ability of the approach for other PAHs. It would be necessary to point out that the determination of the optimal condition for VisNIR spectroscopic method and the effects of the treatment levels on the prediction ability of the approach for phenanthrene is outside the scope of the present paper.

### 3.2. Spectral Reflectance of Diesel-Contaminated Soils

**Figure 2:**
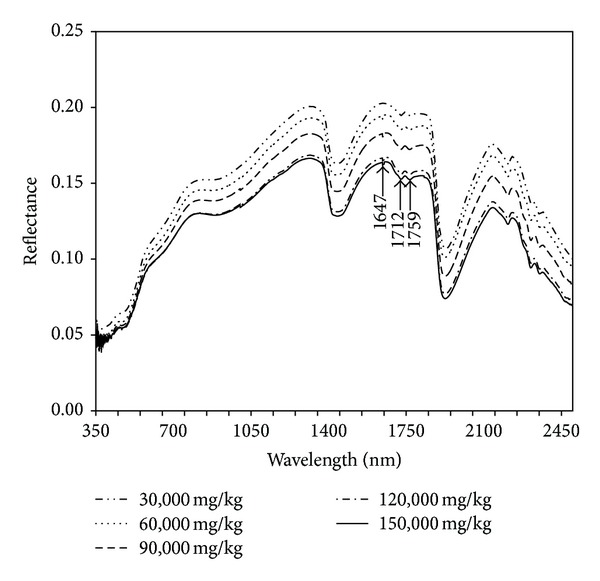
Full-wavelength mean VisNIR spectral reflectance curves of the diesel-contaminated soils.

Figure 2 shows the full wavelength (350 to 2500 nm) mean VisNIR spectral reflectance curves of the diesel-contaminated soils. As can be seen from the figure, soil spectral reflectance decreased with the increasing diesel concentration particularly in the NIR region (700–2500 nm). Spectral absorption minima of hydrocarbon-based oil are apparent around 1647, 1712, and 1759 nm in the first overtone region of the NIR band (Figure 2). The absorption around 1647 nm is attributed to C–H stretching modes of ArCH [12, 29, 30] likely linked to PAH. Absorptions around 1712 and 1759 nm are attributed to C–H stretching modes of terminal CH_3_ and saturated CH_2_ groups linked to TPH, both present in the contaminating diesel fuel [12, 15, 29, 30].

### 3.3. Accuracy of Prediction of Phenanthrene

Table 3 summarizes the statistical results of PLS models in cross-validation and prediction sets for the prediction of phenanthrene. In this model category, the entire 150 VisNIR spectra were combined including both the treatments and levels in the overall model to predict phenanthrene. Only three outlying samples were removed from the calibration dataset (114 samples). From Table 3, the PLS regression models use six LVs for the first derivative and ten LVs for the reflectance models. These are comparable to the range of 6–8 LVs reported for the prediction of saturates, aromatics, resins, and asphaltenes (SARA) fractions in crude oil [12].

**Figure 3:**
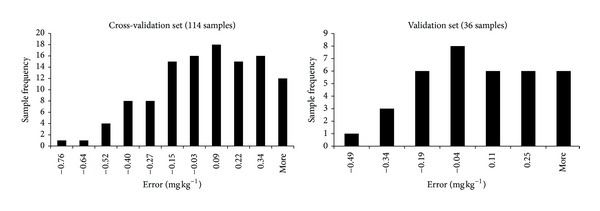
Histogram plot showing the distribution of error between measured and predicted datasets in cross-validation and validation sample sets obtained after PLS regression analysis.

The histogram plot of the error distribution between measured and predicted dataset in cross-validation and validation sample set obtained after PLS regression analysis is shown in Figure 3. The histogram shows that about 75% of the error in cross-validation set is less than 0.34 mg kg^−1^ in absolute values. In the validation set, 72% of the error is less than 0.25 mg kg^−1^ in absolute values. This indicates that the number of samples with low error is relatively larger than the number with high error (Figure 3). The histogram also shows that the error distribution is normally distributed for the validation dataset but slightly skewed for the cross-validation dataset. The skewness in the positive range is largely attributed to some cases of underestimation of the PAH in some samples by VisNIR method (Figure 3). Nonetheless, PLS model prediction ability was classified as excellent (Table 3), demonstrating the possibility of adopting the methodology for quantitative determinations.

### 3.4. Regression Coefficients

**Figure 4:**
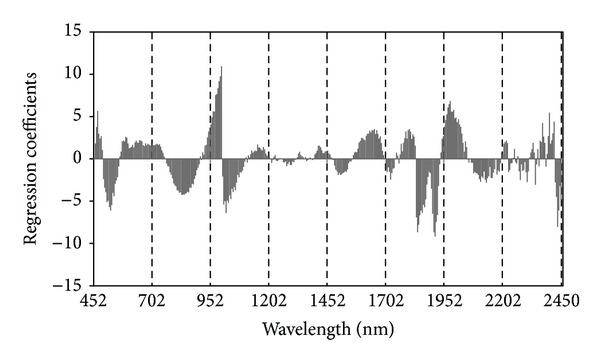
Plot of regression coefficients versus wavelength derived from partial least-squares (PLS) regression analysis for 10 latent variables with raw reflectance spectra of 114 diesel-contaminated soil samples.

In PLS regression analysis, regression coefficients are approximations of model parameters resulting from the linear combination of the predictors. The regression coefficients plot is used to identify important wavelengths for the prediction of relevant soil properties.  Figure 4 shows the bar plot of regression coefficients versus wavelength derived after PLS regression analysis with full-cross-validation for 10 LVs using raw reflectance spectra of 114 calibration samples. In the bar plots, the absolute value of the regression coefficients indicates the relative importance of the wavelength on the basis of explained *X*-variance in the model. Variables with large coefficient play an important role in the model; a positive coefficient shows a positive link to the response, and a negative coefficient shows a negative link [31]. Nonetheless, importance is not restricted to positive coefficients. This plot over the modeling wavelength range of 452–2450 nm shows that the intensities of regression coefficients vary considerably in magnitude (Figure 4).

In the bar plot (Figure 4), negative coefficients around 1712 and 1759 nm (albeit low) show a link to absorptions due to vibrational C–H stretching modes of terminal CH_3_ and saturated CH_2_ functional chemical groups linked to TPH [12, 15]. But positive coefficients around 1647 nm are consistent with absorption bands due to vibrational C–H stretching modes of ArCH functional groups [29, 30] suggesting a link to PAH. These agree with the fact that the spectra of hydrocarbon derivatives originate mainly from combinations or overtones of ArCH functional groups or C–H stretching modes of saturated CH_2_ and terminal CH_3_ groups [12, 15]. In addition to the hydrocarbon absorption bands, large regression coefficients were observed at some other wavelengths in the VisNIR range. This indicates the covariation of PAH with other soil properties having direct spectral responses in the VisNIR range. Soil properties that have direct spectral responses in the NIR range are moisture, clay minerals, and organic carbon, as well as color influence in the visible (Vis) range [32, 33].

Negative coefficients around 497 nm (Figure 4) are linked to blue color absorption band in the Vis range of the spectrum. Reports have shown that changes in soil color are also linked to changes in the amounts of water and/or diesel in the soil [34, 35]. Soil becomes darker with the increase in water content and diesel concentration resulting in an overall increase in absorption or decrease in reflection [34, 35]. Coefficients observed around 950 nm (positive), 1450 nm (positive), and 1950 nm (negative) in the NIR spectrum are absorption bands of water (Figure 4). In the NIR range, O–H stretching modes of water are responsible for the absorptions around 950, 1450, and 1950 nm in the O–H second and first overtones and combinations band, respectively [36]. Coefficients seen around 2200 and 2300 nm (Figure 4) signify absorption features linked to metal-OH bend plus O–H stretch combinations that are characteristic of clay minerals [2, 37]. Negative coefficients can also be seen around 2150 nm (Figure 4), which are absorption features attributed to long-chain C–H + C–H and C–H + C–C stretch combinations unique to soil organic carbon [15]. These absorption features are also conspicuous in the mean VisNIR spectral reflectance curves shown in Figure 2 earlier.

The covariation of clay and organic carbon with PAH is particularly important in the absorption and reflection of PAH (including phenanthrene) in contaminated soils. As stated, PAHs exhibit strong hydrophobicity and largely sorb to the organic carbon in the soil [2]. Dexter et al. [21] suggested that it is not the total amount of organic carbon that controls soil physical behaviour but the amount of complexed and noncomplexed organic carbon (COC and NCOC, resp.). The NCOC is present in soil only if the Dexter index, *n* = clay/organic  carbon, is less than 10 [21]. It is also reported that the NCOC has a higher sorption affinity for PAH than the COC [38]. The Dexter *n* deduced for each soil texture before contamination is shown in Table 1. For all soil texture used in this study, Table 1 shows that only COC was present (as *n* > 10) in all of them. As a result, a significant proportion of PAH is not sorbed to soil because of the absence of NCOC and low PAH sorption affinity of COC. This may explain the amount of NIR spectral signal of sorbed phenanthrene (a PAH) detected. On the other hand, it could be suggested that the presence of NCOC in soil, if *n* < 10, would immobilize PAH within the soil matrix, which might cause increased soil absorption and reduced soil reflection. The swelling characteristics of clay are due to clay minerals, which is such that the higher the clay content, the larger the water retention capacity [9, 32, 34] and the lower the soil diffuse reflectance [39]. Thus, mineralogy of clay may also play a role in controlling the mobility of PAH. The interrelationship between clay and organic carbon may be useful for evaluating the presence of PAH in soils. Presently, the tendency for clay-organic carbon interactions to partly dictate the behaviour of PAH sorption to soil is not well-known, even by conventional laboratory methods, and requires further investigation [38]. Since studies on the application of VisNIR spectroscopy in the determination of PAH in soils are few in the literature [23, 35], there is a dearth of information on the effect of organic matter naturally occurring in the soil on the measurement/prediction of the presence of PAH contamination in soils with VisNIR.

## 4. Conclusions

In the current study, results confirmed the following conclusions: (1) phenanthrene could be predicted with reasonable accuracy (RPD = 2.0–2.32, RMSE = 0.21–0.25 mg kg^−1^, and *r* ^2^ = 0.75–0.83) by VisNIR spectroscopy and (2) the interrelationship between clay and organic carbon may explain the intensity of NIR spectral signal of sorbed PAH in soil. Therefore, the clay-organic carbon interactions may be useful for evaluating the presence of PAH in soils by VisNIR spectroscopy. However, the unique spectral signal of sorbed phenanthrene observed in the current study requires thorough investigation in relation to providing evidence for risk assessment and remediation of contaminated sites.

## Figures and Tables

**Table 1:** Sampling sites and background amount of selected soil properties.

Farm name	Farm location^a^	WRB order^b^	USDA soil textural classification	Clay content (%)	Soil organic carbon (%)	Moisture content (%)	*n* ^c^
College Farm, Silsoe	52°00′30′′N, 0°26′54′′W	Arenosols	Sandy-loam	9	0.76	15.41	11.8
College Farm, Silsoe	52°00′32′′N, 0°26′49′′W	Cambisols	Clay-loam	20	1.89	9.04	10.6
College Farm, Silsoe	52°00′01′′N, 0°26′36′′W	Cambisols	Sandy-clay-loam	35	2.04	15.05	17.2
College Farm, Silsoe	52°00′34′′N, 0°25′60′′W	Cambisols	Loamy-sand	26	1.15	16.13	22.6
Duck End Farm, Wilstead	52°05′08′′N, 0°27′10′′W	Luvisols	Clay	74	1.63	11.91	45.4

^a^Google Earth.

^b^World Reference Base (WRB) classification.

^c^ *n*, Dexter index = clay content/soil organic carbon [21].

**Table 2:** Summary of calibration results for phenanthrene obtained by partial least-squares (PLS) cross-validation analysis carried out with spectra and chemical variables of 25 samples for various concentrations of diesel and moisture and clay contents.

Diesel conc. (mg kg^−1^)	Reflectance spectra					Category^a^	First derivative spectra					Category^a^
	*r* ^2^	RMSE (mg kg^−1^)	SD	RPD	LV		*r* ^2^	RMSE (mg kg^−1^)	SD	RPD	LV	
30,000	0.86	0.11	0.30	2.77	2	A	0.84	0.12	0.30	2.53	2	A
60,000	0.75	0.18	0.37	2.06	2	A	0.74	0.19	0.37	1.97	2	B
90,000	0.89	0.17	0.54	3.11	4	A	0.93	0.14	0.54	3.88	4	A
120,000	0.50	0.36	0.51	1.42	2	B	0.46	0.38	0.51	1.36	2	C
150,000	0.81	0.20	0.46	2.33	2	A	0.77	0.22	0.46	2.11	2	A
Field-moist^b^	0.90	0.16	0.52	3.18	6	A	0.86	0.18	0.52	2.85	6	A

^a^Category of prediction (cross-validation) is the ability of PLS regression analysis for parameter validation and prediction.

Criteria: excellent (A) if RPD > 2.0, almost good (B) if 1.4 ≤ RPD < 2.0, and unreliable (C) if RPD < 1.4 [27].

^b^Field-moist (moisture content = 9.04–16.13%; clay content = 9–74%; diesel concentration = 30,000–150,000 mg kg^−1^).

LV: latent variable; RMSE: root-mean-square error; RPD: residual prediction deviation; SD: standard deviation.

**Table 3:** Sample statistics and results of partial least-squares (PLS) models for the prediction of phenanthrene in cross-validation and prediction datasets for diesel-contaminated soil samples by visible and near-infrared (VisNIR) spectroscopy.

Variable statistics						Model quality									
No. of samples	Min. (mg kg^−1^)	Max. (mg kg^−1^)	Mean (mg kg^−1^)	SD	No. of outliers removed	Reflectance spectra				Category^a^	First derivative spectra				Category^a^
						*r* ^2^	RMSE (mg kg^−1^)	LV	RPD		*r* ^2^	RMSE (mg kg^−1^)	LV	RPD	
Cross-validation set (76%)															

114	0.58	2.49	1.18	0.48	3	0.65	0.28	10	1.71	B	0.62	0.30	6	1.63	B

Prediction set (24%)															

36	0.63	2.20	1.40	0.50	N/A	0.83	0.21	10	2.32	A	0.75	0.25	6	2.00	A

^a^Category of prediction is the ability of PLS regression analysis for parameter validation and prediction. A if RPD > 2.0, B if 1.4 ≤ RPD < 2.0, and C if RPD < 1.4 [27].

LV: latent variable; N/A: not applicable; RMSE: root-mean-square error; RPD: residual prediction deviation; SD: standard deviation.

## Abbreviations

MC: Moisture content
PAH: Polycyclic aromatic hydrocarbon
PLS: Partial least-squares
RMSE: Root-mean-square error
RPD: Residual prediction deviation
TPH: Total petroleum hydrocarbon
VisNIR: Visible and near-infrared.

## References

[1] Latimer JS, Zheng J, Douben PET (2003). The sources, transpojhmrt and fate of PAHs in the marine environment. *PAHs: An Eco-Toxicological Perspective*.

[2] Huang W, Peng P, Yu Z, Fu J (2003). Effects of organic matter heterogeneity on sorption and desorption of organic contaminants by soils and sediments. *Applied Geochemistry*.

[3] Hites RA, Gschwend PM, Cooke M, Dennis AJ, Fisher FL (1982). The ultimate fate of polycyclic aromatic hydrocarbons in marine and lacustrine sediments. *Poly-Nuclear Aromatic Hydrocarbons—Physical and Biological Chemistry*.

[4] Yunker MB, Macdonald RW (1995). Composition and origins of polycyclic aromatic hydrocarbons in the Mackenzie River and on the Beaufort sea shelf. *Journal of Arctic Institute of North America*.

[5] Okoro D, Ikolo AO (2007). Sources and compositional distribution of polycyclic aromatic hydrocarbons in soils of Western Niger Delta. *Journal of Applied Science and Technology*.

[6] Brassington KJ, Pollard SJT, Coulon F, Timmis KN (2010). Weathered hydrocarbon wastes: a risk management primer. *Handbook of Hydrocarbon and Lipid Microbiology*.

[7] Okparanma RN, Mouazen AM (2013). Determination of total petroleum hydrocarbon (TPH) and polycyclic aromatic hydrocarbon (PAH) in soils: a review of spectroscopic and non-spectroscopic techniques. *Applied Spectroscopy Reviews*.

[8] CL:AIRE (2010). Bioremediation of heavy hydrocarbons—reducing uncertainty in meeting risk-based targets: laboratory to field scale (PROMISE Project). *Contaminated Land: Applications in Real Environments (CL:AIRE) Research Bulletin*.

[9] Stenberg B (2010). Effects of soil sample pretreatments and standardised rewetting as interacted with sand classes on Vis-NIR predictions of clay and soil organic carbon. *Geoderma*.

[10] Pasquini C (2003). Near infrared spectroscopy: fundamentals, practical aspects and analytical applications. *Journal of the Brazilian Chemical Society*.

[11] Naes T, Isaksson T, Fearn T, Davies T (2002). *A User Friendly Guide to Multivariate Calibration and Classification*.

[12] Aske N, Kallevik H, Sjöblom J (2001). Determination of saturate, aromatic, resin, and asphaltenic (SARA) components in crude oils by means of infrared and near-infrared spectroscopy. *Energy and Fuels*.

[13] Graham KN (1998). *Evaluation of analytical methodologies for diesel fuel contaminants in soil [M.S. thesis]*.

[14] Malley DF, Graham KN, Webster GRB (1999). Analysis of diesel-contaminated soils by near-infrared reflectance spectrometry and solid phase micro-extraction–gas chromatography. *Journal of Soil Contamination*.

[15] Forrester S, Janik L, McLaughlin M An infrared spectroscopic test for total petroleum hydrocarbon (TPH) contamination in soils.

[16] Forrester ST, Janik LJ, McLaughlin MJ, Soriano-Disla JM, Stewart R, Dearman B (2013). Total petroleum hydrocarbon concentration prediction in soils using diffuse reflectance infrared spectroscopy. *Soil Science Society of America Journal*.

[17] Chakraborty S, Weindorf DC, Morgan CLS (2010). Rapid identification of oil-contaminated soils using visible near-infrared diffuse reflectance spectroscopy. *Journal of Environmental Quality*.

[18] Chakraborty S, Weindorf DC, Zhu Y (2012). Spectral reflectance variability from soil physicochemical properties in oil contaminated soils. *Geoderma*.

[19] Schwartz G, Ben-Dor E, Eshel G (2012). Quantitative analysis of total petroleum hydrocarbons in soils: comparison between reflectance spectroscopy and solvent extraction by 3 certified laboratories. *Applied Environmental Soil Science*.

[20] Bray JG, Viscarra Rossel RA, McBratney AB, Viscarra Rossel RA, McBratney AB, Minasny B (2010). Diagnostic screening of urban soil contaminants using diffuse reflectance spectroscopy. *Proximal Soil Sensing*.

[21] Dexter AR, Richard G, Arrouays D, Czyz EA, Jolivet C, Duval O (2008). Complexed organic matter controls soil physical properties. *Geoderma*.

[22] British Standard Institute (1995). *Determination of Organic and Total Carbon after Dry Combustion (Elementary Analysis)*.

[23] Risdon GC, Pollard SJT, Brassington KJ (2008). Development of an analytical procedure for weathered hydrocarbon contaminated soils within a UK risk-based framework. *Analytical Chemistry*.

[24] Martens H, Naes T (1989). *Multivariate Calibration*.

[25] Tekin Y, Tumsavas Z, Mouazen AM (2012). Effect of moisture content on prediction of organic carbon and pH using visible and near-infrared spectroscopy. *Soil Science Society of America Journal*.

[26] Williams PC, Sobering DC (1986). Attempts at standardization of hardness testing of wheat—II. The near-infrared reflectance method. *Cereal Foods World*.

[27] Chang C-W, Laird DA, Mausbach MJ, Hurburgh CR (2001). Near-infrared reflectance spectroscopy—principal components regression analyses of soil properties. *Soil Science Society of America Journal*.

[28] Viscarra Rossel RA, Walvoort DJJ, McBratney AB, Janik LJ, Skjemstad JO (2006). Visible, near infrared, mid infrared or combined diffuse reflectance spectroscopy for simultaneous assessment of various soil properties. *Geoderma*.

[29] Osborne BG, Fearn T, Hindle PH (1993). *Practical NIR Spectroscopy—With Applications in Food and Beverage Analysis*.

[30] Workman Jr J, Weyer L (2008). *Practical Guide to Interpretive Near-Infrared Spectroscopy*.

[31] CAMO Software (2012). *Interpreting PLS Plots*.

[32] Stenberg B, Viscarra Rossel RA, Mouazen AM, Wetterlind J (2010). Visible and near infrared spectroscopy in soil science. *Advances in Agronomy*.

[33] Kuang B, Mahmood HS, Quraishi MZ, Hoogmoed WB, Mouazen AM, van Henten EJ (2012). Sensing soil properties in the laboratory, in situ, and on-line. A review. *Advances in Agronomy*.

[34] Mouazen AM, de Baerdemaeker J, Ramon H (2005). Towards development of on-line soil moisture content sensor using a fibre-type NIR spectrophotometer. *Soil and Tillage Research*.

[35] Okparanma RN, Mouazen AM (2013). Combined effects of oil concentration, clay and moisture contents on diffuse reflectance spectra of diesel-contaminated soils. *Water Air and Soil Pollution*.

[36] Whalley WR, Stafford JV (1992). Real-time sensing of soil water content from mobile machinery: options for sensor design. *Computers and Electronics in Agriculture*.

[37] Viscarra Rossel RA, McGlynn RN, McBratney AB (2006). Determining the composition of mineral-organic mixes using UV-vis-NIR diffuse reflectance spectroscopy. *Geoderma*.

[38] Soares AA, Moldrup P, Minh LN, Vendelboe AL, Schjonning P, de Jonge LW (2013). Sorption of phenanthrene on agricultural soils. *Water Air and Soil Pollution*.

[39] Viscarra Rossel RA, McBratney AB (1998). Laboratory evaluation of a proximal sensing technique for simultaneous measurement of soil clay and water content. *Geoderma*.

